# Schwartz Formula: Is One *k*-Coefficient Adequate for All Children?

**DOI:** 10.1371/journal.pone.0053439

**Published:** 2012-12-28

**Authors:** Vandrea Carla De Souza, Muriel Rabilloud, Pierre Cochat, Luciano Selistre, Aoumeur Hadj-Aissa, Behrouz Kassai, Bruno Ranchin, Ulla Berg, Maria Herthelius, Laurence Dubourg

**Affiliations:** 1 Centre de Référence des Maladies Rénales Rares, Service de Néphrologie et Rhumatologie Pédiatriques, Hospices Civils de Lyon, Lyon, France; 2 Exploration Fonctionnelle Rénale et Métabolique, Groupement Hospitalier Edouard Herriot, Hospices Civils de Lyon, Lyon, France; 3 Universidade de Caxias do Sul, Centro de Ciências da Saúde, Caxias do Sul, Brazil; 4 Université Claude-Bernard, Lyon, France; 5 Hospices Civils de Lyon, Service de Biostatistique, Lyon, France; 6 CNRS, UMR5558, Laboratoire de Biométrie et Biologie Evolutive, Equipe Biostatistique-Santé, Villeurbanne, France; 7 FRE 3310, CNRS, Université Claude-Bernard, Lyon, France; 8 Pontifícia Universidade Católica do Rio Grande do Sul, Departamento de Nefrologia, Porto Alegre, Brazil; 9 Inserm CIC 201, EPICIME, UMR 5558, Service de Pharmacologie Clinique, Lyon, France; 10 Department of Clinical Science, Intervention and Technology, Division of Pediatrics, Karolinska Institutet, Karolinska University Hospital Huddinge, Stockholm, Sweden; University of São Paulo School of Medicine, Brazil

## Abstract

**Background/Objective:**

Plasma-creatinine-based equations to estimate the glomerular filtration rate are recommended by several clinical guidelines. In 2009, Schwartz *et al*. adapted the traditional Schwartz equation to children and adolescents but did not find different k-coefficients between children and adolescents (k = 36.5 for all patients). We reevaluated the coefficient of the 2009-Schwartz formula according to sex and age in a pediatric population.

**Patients/Methods:**

We used linear mixed-effects models to reestimate the 2009-Schwartz k-coefficient in 360 consecutive French subjects aged 1 to 18 years referred to a single centre between July 2003 and July 2010 (965 measurements). We assessed the agreement between the estimated glomerular filtration rate obtained with the new formula (called Schwartz-Lyon) and the rate measured by inulin clearance. We then compared this agreement to the one between the measured glomerular filtration rate and 2009-Schwartz formula, first in the French then in a Swedish cohort.

**Results:**

In Schwartz-Lyon formula, k was estimated at 32.5 in boys <13 years and all girls and at 36.5 in boys aged ≥13 years. The performance of this formula was higher than that of 2009-Schwartz formula in children <13 years. This was first supported by a statistically significant reduction of the overestimation of the measured glomerular filtration rate in both cohorts, by better 10% and 30% accuracies, and by a better concordance correlation coefficient.

**Conclusions:**

The performance and simplicity of Schwartz formula are strong arguments for its routine use in children and adolescents. The specific coefficient for children aged <13 years further improves this performance.

## Introduction

Assessing the renal function is of utmost importance for the diagnosis and prognosis of kidney diseases. Up to now, glomerular filtration rate (GFR) has been the most widely used index in clinical practice. However, determining the true GFR is cumbersome, costly, and difficult to perform in everyday medical practice. [1] The use of a GFR estimating equation as a noninvasive alternative has been recommended by the clinical guidelines on Chronic Kidney Disease (CKD) management [2], [3] and many formulas based on plasma creatinine (PCr) have been proposed, both for adult and pediatric populations. [4] In 2002, the Clinical Practice Guidelines of the National Kidney Foundation Kidney Disease Outcomes Quality Initiative (NKF KDOQI) recommended the use of the formulas proposed by Schwartz *et al*. (1976) or Counahan-Barratt *et al*. (1976) for the estimation of GFR in children. [2].

In fact, PCr is the central component of these formulas; thus, the accuracy of GFR estimation depends highly on the method chosen for PCr measurement. Recently, important improvements in laboratory techniques have permitted: 1) to improve the specificity of creatinine measurement (using a kinetic colorimetric compensated Jaffé technique or enzymatic methods) and 2) standardization by the isotope dilution mass spectrometry (IDMS) method. [5], [6] With these improvements, the results became logically lower than those given by the classical Jaffé reaction; all formulas established with previous PCr measurement methods are no more adequate and should be revised in several respects. [7].

For example, childhood and adolescence are dynamic periods of development with rapid changes in body size, shape, and composition, which influence muscle mass and, consequently, creatinine production. In 2009, using an enzymatic PCr assay, Schwartz *et al*. adapted the traditional Schwartz equation to children and adolescents [8] but did not demonstrate *k-*coefficient changes with puberty (k = 36.5 for all patients) probably because the studied population was suffering from mild to severe CKD and notable growth retardation (height averaged at the 22.8 percentile (range 5.5–51.3)).

The aims of the present study were: 1) to reestimate new k-coefficients of the 2009-Schwartz equation according to sex and age and assess the performance of the new equation (herein called “Schwartz-Lyon”) in a French cohort of children and adolescents with mild to moderate CKD and no significant growth retardation; and 2) to validate the revised formula on a different cohort.

## Patients and Methods

### The French cohort

This cohort consisted of 360 consecutive patients aged 1 to 18 years, who were referred to the Renal and Metabolic Function Exploration Unit at Edouard Herriot Hospital (Lyon, France) between July 2003 and July 2010 for measurement of inulin clearance because of suspected or established renal dysfunction. A written informed consent was obtained from all patients or their families prior to measurement of the renal clearance of inulin. The consent form contained information on to the procedure itself as well as on the later use of the information in research works. According to the French Law, concerning the use of a database without direct identification of patients, it was not necessary to obtain an ethical approval (law 2006–450, april 19th 2004; Commission nationale de l’informatique et des libertés -CNIL).

Heights, weights, and ages were recorded. Weights and heights were expressed in percentiles according to the CDC’s sex appropriate weight-for age and length or stature-for-age growth charts. [9] Growth retardation was defined by a length/stature below the 3^rd^ percentile on the growth charts.

This retrospective cohort was divided into two age groups: 218 children aged <13 years and 142 adolescents aged 13 to 18 years. The age limits for adolescents were chosen according to the original work of Schwartz *et al*. [10] Each participant may have contributed several measurements. However, all children simultaneous measurements of inulin clearance and plasma creatinine were made before age 13 and all adolescent simultaneous measurements were made from 13 to 18 years old.

### The Swedish cohort

Data on 109 patients were obtained from the Pediatric Nephrology Unit at Karolinska University Hospital in Huddinge (Stockholm, Sweden). The Swedish nephrology unit used the same methods for renal evaluations (inulin clearance and creatinine measurement) as the French center. [11], [12].

### Measurement of Plasma Creatinine

PCr was obtained with a kinetic colorimetric compensated Jaffé technique (Roche Modular, Meylan, France). To assess the stability of the PCr assay along the study period, Blinded ProBioQal controls were carried out every five weeks and a nationwide-blinded control was carried out each year. The intra-assay coefficient of variation was around 0.7%. The inter-assay coefficients of variation at low (45–60 µmol/L) and high (580 µmol/L) plasma creatinine concentrations were around 4% and 1.5%, respectively. [13].

All PCr measurements were performed with the same method over the whole study period. These measurements were standardized by linear regression adjustment of the concentrations obtained by the compensated Jaffé assay and the concentrations obtained by liquid chromatography-mass spectrometry (LCMS).

The calibration equation was: Standardized serum creatinine = 0.9395 * (Jaffé compensated serum creatinine in µmol/L) +4.6964. The intercept (4.6964; 95% confidence interval (CI) [−2.4619 to 11.8656]) and the slope (0.9395; 95% CI [0.8719 to 1.0072]) of the calibration line were not significantly different from 0 and 1, respectively. The coefficient of correlation *r* was 0.97. The mean difference between the LCMS and the compensated Jaffé values was 1.24±10.05 µmol/L.

### Measurement of GFR

The GFR was measured (mGFR) by the renal clearance of inulin (polyfructosan infusion, Inu test®, Fresenius Kagi, Graz, Austria). A standard technique was used by a trained staff with a continuous infusion after a 30 mg/kg priming dose of polyfructosan. Water diuresis was induced by oral administration of 5 mL/kg of water followed by 3 mL/kg every 30 minutes combined with an intravenous infusion of 0.9% sodium chloride. Patients needing intermittent urethral catheterization were excluded from this study. The measurements of plasma and urine polyfructosan were performed using the same enzymatic method (Inu test®). The results were expressed per 1.73 m^2^ body surface area (Dubois formula: BSA = height^0.725^ * weight^0.425^ * 0.007184). [14].

### Statistical Analysis

#### Estimation of the k-coefficient of Schwartz-Lyon formula in the French cohort

A linear mixed regression was used to model the clearance of inulin according to the ratio of height over PCr with an intercept fixed at zero and a random effect to take into account the correlation between clearance measurements made in the same patient. [15], [16] A model was built for children aged <13 years and another for adolescents. Considering that puberty induces the main body composition changes between boys and girls, an interaction term between sex and the height/PCr ratio was added to the adolescent model. A modeling of the residual variance using a power function of the predicted mean allowed correcting for heteroscedasticity of the residuals.

The *lme* function of package nlme in R software was used to fit the model and the restricted maximum likelihood (REML) method was used to estimate the model parameters. [16].

#### Performance of 2009-Schwartz and Schwartz-Lyon formulas in the French and Swedish cohorts

We assessed the agreement first between the mGFR and the eGFR as estimated with 2009-Schwartz formula, then between the mGFR and the eGFR as estimated with Schwartz-Lyon formula in the French and in the Swedish cohort.

Each mGFR-eGFR agreement was assessed using the following tools:

1. The mean ratio = mean eGFR/mGFR to assess bias. Here, the ratio was preferred to the difference between eGFR and mGFR in order to correct for heteroscedasticity.
2. The standard deviation of the eGFR/mGFR ratio to assess the heterogeneity of this ratio.
3. Bland and Altman graphs.
4. The Concordance Correlation Coefficient (CCC) between each eGFR and the mGFR (after logarithmic transformation of their values). The CCC is a measure of agreement that adjusts the Pearson correlation coefficient downward whenever there is a systematic bias between the methods being compared. [17], [18]
5. The 10% and 30% accuracies according to the KDOQI guidelines. [2] These are defined as the proportions of the estimates falling respectively within the interval mGFR±10% or the interval mGFR±30%.

The comparisons of the eGFR mean ratios, the CCCs, and the 10 and 30% accuracies between the two formulas used, respectively, a paired *t* test, the bootstrap 95% confidence intervals of the difference between the two CCCs, and McNemar’s test.

In the French cohort, a random intercept model was used to estimate the mean ratio and the standard deviation of the ratio. This allowed for repeated measurements in the same patients and estimates of intra-patient and inter-patient variances.

Bland & Altman graphs were built using the mGFR values on the x-axis because the mGFR (i.e., clearance) is considered as the gold standard method for GFR measurement. [19], [20] In fact, Krouwer has shown that the means of two methods being compared should not be used on the X axis whenever one of the methods is the gold standard, but that it is the gold standard that should be used instead. [20].

#### Formula performance by CKD stage in the French cohort

The mean eGFR/mGFR ratio, the standard deviation of the ratio, and the 10% and 30% accuracies were assessed in each of the three CKD stages (Stage 1: GFR >90 mL/min/1.73 m^2^, Stage 2∶60≤ GFR <90 mL/min/1.73 m^2^, Stage 3: GFR <60 mL/min/1.73 m^2^).

The ability of the formulas to distinguish between patients without CKD and patients with different-stage CKD was estimated using the area under the ROC curve. The Delong-Clarke-Pearson method was used to compare the AUCs given by the two formulas.

All the analyses were performed using R for windows version 2.13. A value of p<0.05 was considered for statistical significance.

## Results

### Clinical Characteristics of the French Cohort

The French cohort of children and adolescents included 360 patients of whom 53% were males. These patients contributed 965 measurements (an average of 3 mGFR and PCr measurements per patient). In this cohort, 28% of the patients had a mGFR <60 mL/min/1.73 m^2^ (Table 1).

**Table 1:** Characteristics of the French and the Swedish cohort of children and adolescents.

Characteristics	French cohort (1–17.9 years)	Swedish cohort (4–17.9 years)
Patients	360	109
Males	190	55
Age (yr, median [IQR])	12.7 [9.5–15.3]	13.7 [11.2–16.2]
<13 years (n)	218	41
13–17.9 years (n)	142	68
Weight (kg, median [IQR])	38.0 [28.0–50.5]	50.2 [37.7–62.4]
Weight percentile (median [IQR])	33.4 [14.2–60.0]	65.4 [40.3–89.4]
Height (cm, median [IQR])	147.0 [131.0–159.0]	153.8 [142.4–162.4]
Height percentile (median [IQR])	27.1 [7.2–57.9]	40.3 [18.7–73.2]
BSA (m^2^, median [IQR])	1.28 [1.02–1.51]	1.46 [1.23–1.68]
BMI (kg/m^2^, median [IQR])	17.5 [15.8–20.0]	20.1 [18.1–23.8]
PCr (µmol/L, median [IQR])	58.0 [45.0–75.5]	92.5 [54.2–171.2]
mGFR (mL/min/1.73 m^2^, median [IQR])	86.0 [65.0–109.0]	62.0 [33.2–91.0]
Main Diagnosis, n (%)		
Renal transplant	76 (21)	35 (32)
Other organ transplant	65 (18)	0
Glomerular disease	49 (14)	29 (27)
Chronic Kidney disease	17 (5)	19 (17)
Systemic disease	54 (15)	0
Miscellaneous	99 (27)	26 (24)
Number of mGFR	965	109
KDOQI classification, n (%)		
I	431 (46)	28 (26)
II	337 (35)	33 (30)
III	173 (18)	22 (20)
IV	13 (1)	19 (17)
V	2 (<1)	7 (7)

BSA =  body surface area, BMI = body mass index, IQR = interquartile range, mGFR = glomerular filtration rate measured by inulin clearance.

### Estimation of the New Coefficients According to Sex and Age

Applying a linear mixed-effects model to children <13 years old, we found a regression coefficient (standard error) of 32.5 (0.33).

The use of the same model in children ≥13 years led to a higher coefficient in boys than in girls: the regression coefficient (standard error) was 36.5 (0.55) in boys and 32.5 (0.74) in girls.

### Performance of Schwartz-Lyon Formula in the French Cohort

2009-Schwartz and Schwartz-Lyon formulas were applied to the French cohort (Table 2). The performance of Schwartz-Lyon was better than that of 2009-Schwartz (Table 3) in the whole cohort, in children alone, and in adolescents alone. Indeed, Schwartz-Lyon formula had a lower bias (as shown by mean ratios and CCCs significantly closer to 1) than those with the 2009-Schwartz formula (Table 3). The standard deviations of the ratio were slightly lower with the Schwartz-Lyon formula and the 30% accuracies of Schwartz-Lyon formula were significantly higher than those of the 2009-Schwartz formula. This illustrates a better agreement between Schwartz-Lyon formula and mGFR than between 2009-Schwartz and mGFR (Table 3).

**Table 2:** Formulas used for estimating the glomerular filtration rates.

Name	Formula
2009-Schwartz	eGFR = *k* * height/PCr*k* = 36.5
Schwartz-Lyon	eGFR = *k* * height/PCr*k* = 36.5 in males aged >13 years*k* = 32.5 in others

Height is expressed in cm. PCr = Plasma creatinine, expressed in µmol/L.

**Table 3:** Agreement between formulas and inulin clearance according to age subgroup in the French and the Swedish cohorts.

	French cohort			Swedish cohort	
	2009-Schwartz	Schwartz-Lyon		2009-Schwartz	Schwartz-Lyon
All					
Mean ratio ± SD	1.07±0.22	1.02* ±0.21		1.03±0.23	0.96±0.19
10% accuracy (%)	38	41*		39	37
30% accuracy (%)	84	89*		85	91
CCC (95% CI)	0.81 (0.79–0.83)	0.83 (0.81–0.85)		0.95 (0.93–0.97)	0.96 (0.94–0.97)
ρ×Cb	0.82×0.98	0.83×0.99		0.95×0.99	0.94×0.97
Children (<13 yrs)					
Mean ratio ± SD	1.10±0.20	1.02* ±0.19		1.15±0.25	1.03* ±0.22
10% accuracy (%)	40	45*		34	41
30% accuracy (%)	83	89*		80	85
CCC (95% CI)	0.84 (0.80–0.87)	0.87* (0.83–0.90)		0.94 (0.90–0.97)	0.96 (0.92–0.98)
ρ×Cb	0.87×0.96	0.87×0.99		0.95×0.98	0.96×0.99
Adolescents (13–17.9 yrs)					
Mean ratio ± SD	1.02±0.23	1.01* ±0.22		0.96±0.19	0.92±0.16
10% accuracy (%)	36	37		39	33
30% accuracy (%)	85	88*		86	94
CCC (95% CI)	0.85 (0.80–0.89)	0.86* (0.82–0.90)		0.95 (0.93–0.97)	0.95 (0.93–0.97)
ρ×Cb	0.86×0.98	0.86×0.99		0.95×0.99	0.96×0.98

CCC = concordance correlation coefficient (CCC = ρ×Cb), ρ = Pearson coefficient, CI = Confidence Interval, Cb = factor for Pearson coefficient correction in CCC - *p<0.05 between formulas.

The two formulas underestimated the mGFR in patients with normal renal function (Table 4). But, in patients with renal insufficiency, the mGFR was overestimated with the 2009-Schwartz formula and this overestimation increased along with the severity of the disease. This overestimation was significantly lower with Schwartz-Lyon than with 2009-Schwartz formula (Table 4).

**Table 4:** Agreement between formulas and inulin clearance according to the GFR group in the French cohort and estimation of the diagnostic ability of the two formulas according to the CKD stage.

CKD stage	2009-Schwartz	Schwartz-Lyon
*Stage 3 (GFR<60 mL/min/1.73 m^2^)*		
Mean ratio ± SD	1.20±0.27	1.10* ±0.25
10% accuracy	27	37*
30% accuracy	70	81*
AUC (95% CI) for GFR<60vs. GFR≥90	0.95 (0.94; 0.97)	0.96 (0.95; 0.97)
*Stage 2 (60≥ GFR <90 mL/min/1.73 m^2^)*		
Mean ratio ± SD	1.06±0.20	0.98* ±0.19
10% accuracy	38	41
30% accuracy	85	87
AUC (95% CI) for ≥60 GRF <90vs. GFR≥90	0.91 (0.89; 0.92)	0.92 (0.91; 0.94)
*Stage 1 (GFR>90 mL/min/1.73 m^2^*		
Mean ratio ± SD	0.98±0.19	0.95±0.19
10% accuracy	37	37
30% accuracy	89	89
AUC (95% CI) for GFR<90vs. GFR≥90	0.92 (0.90; 0.94)	0.93* (0.92; 0.95)

* p<0.05 between Schwartz-Lyon formula and 2009-Schwartz - Mean ratio = mean ratio (eGFR/mGFR) value, AUC = area under ROC curves, 95% CI = 95% Confidence Interval.

The abilities of the formulas to distinguish patients with renal insufficiency from subjects with normal renal function was slightly higher with Schwartz-Lyon formula whatever the severity of the renal insufficiency as shown by greater areas under the ROC curves (Table 4). The estimations of the areas under the ROC curves were greater than 90% with the two formulas (Table 4).

### External Validation on the Swedish Cohort

We applied Schwartz-Lyon formula to the Swedish cohort (Table 1). Overall, the two equations showed similar performance in the Swedish and the French population (Table 3).

**Figure 1:**
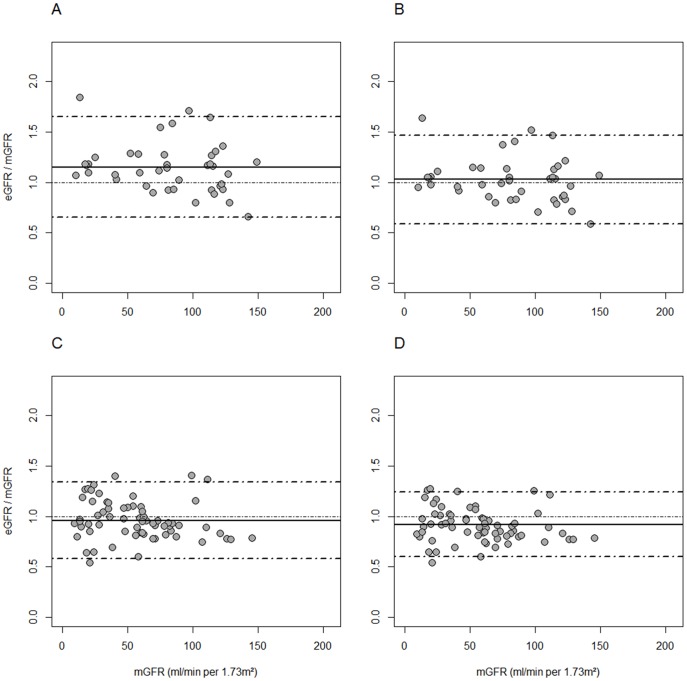
Bland and Altman plots showing the ratio estimated GFR/measured GFR versus the measured GFR as gold standard in the Sweden cohort. Panel A: 2009-Schwartz in children. Panel B: Schwartz-Lyon in children. Panel C: 2009-Schwartz in adolescents. Panel D: Schwartz-Lyon in adolescents.

In the Swedish population, the bias of Schwartz-Lyon formula was significantly lower than that of 2009-Schwartz among children (mean ratio closer to 1) (Table 3 and Figure 1). We did not find statistically significant differences between the two formulas regarding the 10% accuracy, the 30% accuracy, or the CCC in the children group, probably due to the small number of patients in this age group (41 patients <13 years). However, Schwartz-Lyon formula underestimated the mGFR by 8% among the adolescents (mean eGFR/mGFR ratio of 0.92) and overestimated it by 3% in the children whereas 2009-Schwartz formula underestimated the mGFR by 4% in the adolescents and overestimated it by 15% in the children (Table 3).

Bland & Altman graphs show an overestimation of the mGFR at values under 50 mL//min/1.73 m^2^ for the 2009-Schwartz formula in children (all ratios >1) (Figure 1A). This overestimation was reduced with Schwartz-Lyon formula (Figure 1B). We did not find significant differences between the two formulas in the adolescents (Figure 1C and 1D).

## Discussion

Chronic kidney disease and its complications cause substantial morbidity and mortality. [21] Most patients with CKD are identified or treated only after considerable delays and some adults presenting with CKD may have developed early stages of CKD during childhood or adolescence. [22], [23] An early detection of CKD is therefore essential to reduce the CKD-associated cardiovascular morbidity and delay the progression toward end-stage renal disease. eGFR equations facilitate detection, evaluation, and management of the disease, and should result in improved patient care and better clinical outcomes. [24].

The original Schwartz formula [10] developed in children in 1976 has been recently adapted to current methods of PCr assay [8] and its use is now recommended to estimate GFR in children. However, this original formula was developed in a cohort of 349 North American children with mild to severe CKD (median GFR 41 mL/min/1.73 m^2^) and remarkable growth retardation and could not take age into account, especially in adolescent boys. In a previous study on the original Schwartz formula in a small number of patients (n = 167), we determined new k-coefficients using a simple regression method [25] similar to that used for the original formula. The study led to different coefficients according to age and sex (*k* = 33 for boys and girls <13 years old and *k* = 37 for boys ≥13 years old). These coefficients were validated in a pediatric population of 252 patients aged 10.7±4.0 years (range 4.4 to 19.9) with mild or no renal insufficiency (mean mGFR 101±32 mL/min/1.73 m^2^) and no significant growth retardation. [4] Then, taking benefit from a larger number of patients with repeated measurements in our database, we decided to reestimate the former *k*-coefficients using statistical methods that allow for repeated measurements (linear mixed-effects models) in order to obtain much more accurate coefficients according to children age and improve the performance of this creatinine-based GFR estimating equation.

The main results of the present study were: 1) a demonstration of the interest of using different coefficients (one for children <13 years old and female adolescents and another for male adolescents) as previously shown by Schwartz in 1976; 2) the validation of the new coefficients (Schwartz-Lyon formula) in another population of patients.

A comparison between the last Schwartz formula (2009-Schawrtz) and the present Lyon-Schwartz shows several interesting differences. First, the 2009-Schwartz was developed in a cohort of children with mild to severe CKD and notable growth retardation [8] and no age-subgroup subdivision (as in the 1976-formula). [26], [10] The population studied here experienced mild or no renal insufficiency (median GFR 86 mL/min/1.73 m^2^) and less than 10% of it experienced growth retardation. This led to a Schwartz-Lyon *k*-coefficient close to that of the 2009- Schwartz formula for boys >13 years old (*k* = 36.5), but to a lower *k*-coefficient (*k* = 32.5) in all girls and in boys <13. Second, the new Schwartz-Lyon formula elaborated in a French cohort was validated in a Swedish cohort of similar age distribution but devoid of growth retardation and with lower renal function (median GFR 62 mL/min/1.73 m^2^). The new formula showed a good performance with a 30% accuracy of more than 90% (which is the lower limit recommended by the KDOQI clinical practical guidelines) [12] and with a mean underestimation of mGFR of about 4%. Compared to the 2009-Schwartz formula, the new Schwartz-Lyon formula showed a better performance in the children group (mean eGFR/mGFR ratio closer to one, p<0.05) but equivalent performance in the adolescent group. Finally, the 30% accuracy was not significantly higher with Schwartz-Lyon formula than in 2009-Schwartz formula probably because of the limited number of patients in each group. We believe that the use of a specific coefficient in children <13 and girls ≥13 years of age (who have a lower muscle mass than male adolescents) improved slightly but significantly the performance of the formula.

Schwartz-Lyon and 2009-Schwartz formulas tended to underestimate the mGFR in subjects with normal renal function^2^ and overestimate it in patients with renal insufficiency. Previous studies using the 2009-Schwartz equation have shown very similar results. Staples *et al*. [27] compared eGFR with mGFR using iothalamate clearance in a population of 503 children aged 1 to 16 years with mean mGFR of 110.6 mL/min/1.73 m^2^ and found a mean bias of −5.8 and −9.1 mL/min/1.73 m^2^ for mGFR values below and above 90 mL/min/1.73 m^2^, respectively. These results are in agreement with those of Pottel *et al*. [28] and of Fadrowski *et al*. [22] who suggested that the 2009-Schwartz formula may underestimate renal function in patients including 8.9% adolescents with eGFR <75 mL/min/1.73 m^2^. Chavers *et al*. [29] have also found that the CKiD formula (that included PCr, cystatin C, and blood urea nitrogen) underestimated the renal function in undiseased subjects.

Several strengths of the present study can be pointed out: 1) the use of the reference standard method for GFR measurement (i.e., inulin clearance) both in the test and the validation population; 2) the use of an IDMS standardized creatinine for the validation of the coefficients; and, 3) a validation of the new coefficients in a different population of patients (the Swedish cohort). However, the study present also some limitations:1) the age lower-limit for adolescence was arbitrarily set to 13 as in Schwartz’s original study and no objective marker of puberty was used to classify male adolescents (e.g., Tanner stages); 2) unintentionally, the study populations did not include non-Caucasian patients; thus, it could not assess the effect of ethnicity; and 3) the performance of eGFR equations in patients with mGFR <30 mL/min/1.73 m^2^ could not be assessed because of the small number of such patients in the study population.

In conclusion, the present study validated and completed that of Schwartz *et al* in 2009 with a determination and a validation of a specific coefficient for children and female adolescents, which improves slightly the performance of the initial formula. The good performance and the simplicity of 2009-Schwartz and Schwartz-Lyon formulas are strong arguments for recommending them in routine care of children and adolescents.
